# Sarcopenia Predicts Adverse Prognosis in Patients with Heart Failure: A Systematic Review and Meta-Analysis

**DOI:** 10.31083/j.rcm2409273

**Published:** 2023-09-25

**Authors:** Yunyue Liu, Mengyu Su, Yang Lei, Jinping Tian, Lin Zhang, Di Xu

**Affiliations:** ^1^School of Nursing, Nanjing Medical University, 210000 Nanjing, Jiangsu, China; ^2^Department of Cardiology, Nanjing Drum Tower Hospital, 210000 Nanjing, Jiangsu, China

**Keywords:** sarcopenia, heart failure, prognosis, all-cause mortality, major adverse cardiovascular events, meta-analysis

## Abstract

**Background:**

This study aims to assess whether sarcopenia can be used to predict prognosis in patients with heart failure (HF) and if different diagnostic criteria for sarcopenia and diverse regions where studies were conducted could affect prognostic outcomes, thus providing a preliminary basis for early identification and prediction of poor prognosis in HF.

**Methods:**

The PubMed, Cochrane, Embase, and CNKI (China National Knowledge Infrastructure) databases were searched from inception until March 2023. Cohort studies evaluating the prognostic effect of sarcopenia in patients with HF were included. Two authors independently assessed the studies according to the Newcastle-Ottawa Scale. The meta-analyses were performed using RevMan 5.3 software. The study results were reported using a checklist of Preferred Reporting Items for Systematic Reviews and Meta-analyses were used to report the study results.

**Results:**

A total of 12 studies with 3696 HF patients were included. The results showed that the sarcopenia population had a higher risk of all-cause mortality (HR (hazard ratio) = 1.98, 95% CI (confidence interval): 1.61–2.44) and major adverse cardiovascular events (MACE) (HR = 1.24, 95% CI: 1.06–1.45) compared to the non-sarcopenia population. Moreover, the subgroup analysis reported that different diagnostic criteria for sarcopenia and diverse regions were statistically significant for all-cause mortality, except for the Europe subgroup (HR = 1.34, 95% CI: 0.89–2.02). In the subgroup analysis of MACE, all subgroups were statistically significant except for the European Working Group on Sarcopenia in Older People (EWGSOP) (HR = 1.39, 95% CI: 0.86–2.25) and European subgroups (HR = 1.39, 95% CI: 0.86–2.25).

**Conclusions:**

Sarcopenia is associated with poor prognosis, including all-cause mortality and MACE, in patients with HF. However, due to the adoption of various diagnostic criteria in different regions of the world, these results need further validation.

## 1. Introduction

Heart failure (HF) represents the terminal stage of most cardiovascular diseases and has become an increasingly serious public health problem affecting every country worldwide [1]. According to the global epidemiological reports, over 64 million people are affected by HF worldwide. In addition to its significant prevalence, the high rate of morbidity, mortality, and readmission rates among HF patients also have led to substantial health, economic, and social losses [2]. As a progressive and complex syndrome, HF causes symptoms such as dyspnea, weakness, and fluid retention, and reduces health-related quality of life [3, 4, 5].

Sarcopenia is a widespread skeletal muscle disorder characterized by poor physical performance, low muscle strength and loss of muscle mass or quantity. It is closely associated with HF [6, 7]. Firstly, HF and sarcopenia share several common risk factors, including aging, hormonal changes, malnutrition and malabsorption, inflammation and oxidative stress, apoptosis, lack of exercise, and endothelial dysfunction [5]. Secondly, studies have shown that HF may lead to sarcopenia due to decreased exercise capacity caused by reduced peripheral blood flow [8]. Conversely, sarcopenia also promotes the worsening of clinical conditions in HF patients. It causes a deterioration of quality of life, due to the adverse effects of muscle atrophy on exercise intolerance and ventilation inefficiency [9]. Therefore, sarcopenia and HF appear to be closely intertwined, mutually reinforcing the progression and outcome of each other.

Sarcopenia has been identified as a prognostic factor in HF patients in several studies. However, the prognostic impact is inconsistent. Some studies have reported a poor prognosis for sarcopenia in HF patients [10, 11], while others have reported no statistically significant difference between sarcopenia and HF prognosis [12]. In addition, the effect of different diagnostic criteria for sarcopenia and different regions on the prognosis of HF has been inconsistent, and no consensus conclusion has been reached [11, 12]. Currently, there is no uniformity in the prognostic impact of sarcopenia on HF. Two meta-analyses evaluated the incidence of sarcopenia and its association with the prognosis of HF, but these two meta-analyses focused mainly on assessing prevalence rather than prognosis. The number of studies included in the prognostic analysis was insufficient and did not consider the effects of different diagnostic criteria for sarcopenia and different regions, which did not allow for a meta-analysis to draw rigorous conclusions [13, 14].

Many new studies on the relationship between sarcopenia and HF prognosis have emerged in the last two years, but no consistent conclusion has been reached. Therefore, there is a need for an updated meta-analysis based on the available evidence to assess whether sarcopenia could predict the prognosis of HF patients and if different diagnostic criteria for sarcopenia and diverse regions where studies were conducted affect prognostic outcomes. This can contribute to the assessment and management of HF prognosis.

## 2. Methods

The Preferred Reporting Items for Systematic Reviews and Meta-Analyses (PRISMA) guideline [15] (**Supplementary Material 1**) was used to conduct this meta-analysis. The protocol was entered into PROSPERO (CRD42022365509), the International Prospective Register of Systematic Reviews.

### 2.1 Search Strategy

The PubMed, the Cochrane Central Register of Controlled Trials (CENTRAL), Embase, and China National Knowledge Infrastructure (CNKI) databases were systematically searched from inception until March 2023. Search terms incorporated MeSH terms and keywords related to “sarcopenia” OR “sarcopeni*” OR “muscle weakness” OR “muscle atrophy” AND “heart failure” OR “HF” OR “cardiac failure” OR “heart decompensation” OR “myocardial failure” OR “congestive heart failure”. See **Supplementary Material 2** for detailed search strategy. Grey literature was searched using Open Grey. We also manually looked for additional research that was missed by the search strategy in the reference lists of all eligible papers.

### 2.2 Eligibility Criteria

The inclusion criteria were as follows: Participants were patients meeting the diagnostic criteria for HF [16]. The exposure group consisted of all subjects that were diagnosed with sarcopenia according to a certain diagnostic criteria established by a working group on sarcopenia, a certain research, or clinical experience. The prognostic outcome included all-cause mortality and major adverse cardiovascular events (MACE), including cardiac death, HF readmission, and other HF-related adverse events. The study type was cohort studies, and the language of the studies was either English or Chinese. The exclusion criteria were as follows: review, commentary, editorial, and conference abstract were excluded. Studies that did not provide sufficient outcome data were also excluded, as well as those with no full text unavailable.

### 2.3 Study Selection and Data Extraction

To eliminate duplication, all of the obtained records were imported into EndNote (version 20.0, Clarivate Analytics, Philadelphia, PA, USA). Study titles and abstracts were independently reviewed by two researchers (YYL and MYS), and the complete texts of potential studies were retrieved and evaluated by the same two authors. A third reviewer (LZ) was consulted to settle any discrepancies in study selection. Two researchers (YYL and MYS) independently extracted data including study characteristics (author, year, country), study design, study population (sample size, age, gender), sarcopenia (diagnostic criteria, cutoff value, measurement), follow-up duration, and prognostic outcomes. Disagreements between reviewers were settled by conversation or, if necessary, by decision from a third reviewer (LZ).

### 2.4 Quality Assessment

Two authors (YYL and MYS) independently evaluated the included studies using the Newcastle-Ottawa Scale (NOS) criteria for cohort studies [17]. The NOS criteria have a total score of 9 for three dimensions containing eight entries, among which four entries were for population selection (4 points), one entry was for comparability between groups (2 points), and three entries were for exposure (3 points). High-quality studies were defined as those with a score of 5 or higher, with higher scores suggesting a decreased likelihood of bias and higher quality. Any discrepancies were settled in discussion with a third researcher (DX).

### 2.5 Statistical Analysis

The RevMan software, version 5.3 (The Cochrane Collaboration, The Nordic Cochrane Centre, Copenhagen, Denmark), was used to conduct the meta-analyses. The data were summarized using the hazard ratio (HR) and related 95% confidence interval (CI). Cochran’s Q-test and $I^{2}$ index were used to assess the statistical heterogeneity between studies, with $I^{2}$ ≥50% indicating significant heterogeneity. When no significant heterogeneity was detected, the fixed-effects model was applied. The random-effects model was applied otherwise (Higgins & Green, 2019). Sensitivity analysis was carried out to assess the stability and robustness of the results by systematically removing one study on each turn. *p* < 0.05 was considered statistically significant. To assess potential publication bias, funnel plots and Egger tests were performed if the number of included studies was greater than 10.

## 3. Results

### 3.1 Study Selection

**Fig. 1.**
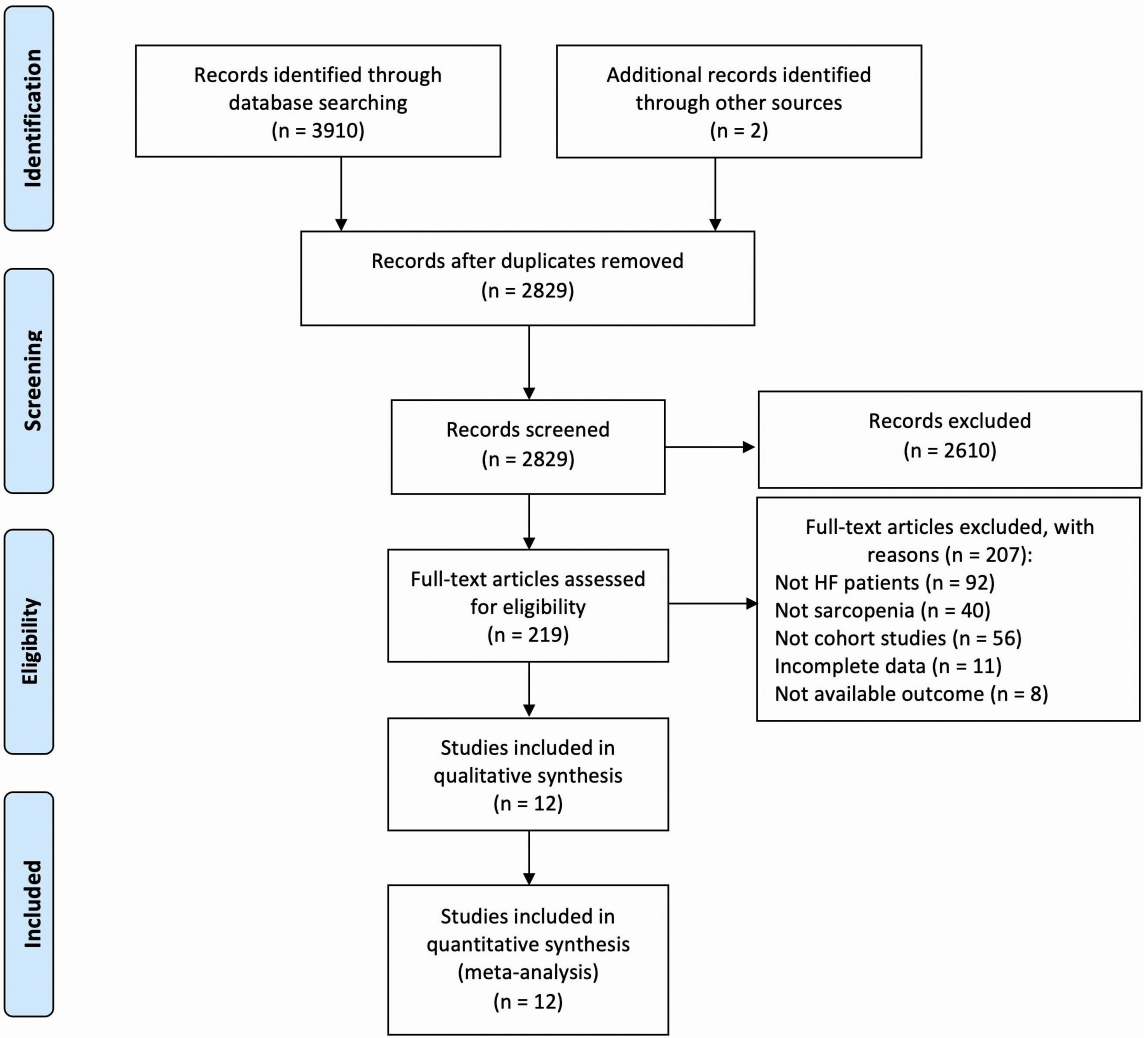
**The PRISMA flow chart of literature screening and selection process**. HF, heart failure.

After deleting duplicates, 2829 publications were left out of the total 3912 publications that were found. 207 articles were removed from the retrieval of 219 articles for additional full-text screening. Ultimately, a total of 12 publications [10, 11, 12, 18, 19, 20, 21, 22, 23, 24, 25, 26] were included in the final review. The PRISMA flow chart for literature screening and selection is shown in Fig. 1.

### 3.2 Study Characteristics

The twelve included studies were all released between 2016 and 2022. The sample size ranged from 58 to 960. There were nine prospective cohort studies and three retrospective cohort studies among the included studies. The majority of the studies (n = 7) were carried out in Japan. Table 1 (Ref. [10, 11, 12, 18, 19, 20, 21, 22, 23, 24, 25, 26]) displays the characteristics of the study. Diagnostic criteria for sarcopenia developed by eight different organizations and research groups were used: the Asian Working Group for Sarcopenia (AWGS) [27], the European Working Group on Sarcopenia in Older People (EWGSOP) [28], EWGSOP2 [29], the Chinese Society of Bone and Mineral Research (CSOBMR) [30], Boutin [31], Ishii [32], Takagi [33], and Harada [26]. Exceptionally, Konishi [24] studied heart failure with preserved ejection fraction (HFpEF) and heart failure with reduced ejection fraction (HFrEF) as two separate groups of patients, and Saito [25] used two different diagnostic methods in the same group of HF patients. To distinguish these data, we named them Konishi A and Konishi B, Saito A, and Saito B, respectively, and included them in the meta-analysis. Diagnostic criteria and cutoff values used to define sarcopenia are shown in Table 2 (Ref. [10, 11, 12, 18, 19, 20, 21, 22, 23, 24, 25, 26]).

**Table 1.** **Characteristics of the included studies**.

Study	Design	Country	Population			Follow-up duration	Outcome
			Sample size	Age (year)	Gender, male (%)		
Onoue 2016 [18]	Prospective cohort study	Japan	HF (n = 119)	76.1 ± 6.2	61	495 days	MACE
Zhou 2017 [19]	Prospective cohort study	China	Chronic HF (n = 182)	77.5 ± 5.9	59.3	36 months	MACE
Nozaki 2019 [20]	Prospective cohort study	Japan	HF (n = 191)	73.3 ± 7.3	71.2	8 months	MACE
Lopez 2019 [10]	Retrospective cohort study	USA	Chronic HF (n = 160)	66.3 ± 13.8	69.4	12 months	All-cause mortality
Von Haehling 2020 [21]	Prospective cohort study	Germany	Chronic HF (n = 268)	67.14 ± 10.86	78.7	67.2 ± 28.02 months	All-cause mortality
Matsumura 2020 [22]	Prospective cohort study	Japan	ADHF (n = 210)	Reduced PMI: 80	Reduced PMI: 48	1.8 years	All-cause mortality
				Preserved PMI: 79	Preserved PMI: 82		
Hu 2020 [23]	Retrospective cohort study	China	HFpEF (n = 240)	Sarcopenia: 69.5 ± 7.1	Sarcopenia: 51.3	30.6 ± 16.7 months	MACE
				Non-sarcopenia: 70.3 ± 9.5	Non-sarcopenia: 55.6		
Konishi A 2021 [24]	Prospective cohort study	Japan	HFpEF (n = 475)	81 ± 7	48.8	12 months	1. All-cause mortality
							2. MACE
Konishi B 2021 [24]	Prospective cohort study	Japan	HFrEF (n = 467)	78 ± 8	68.1	12 months	1. All-cause mortality
							2. MACE
Eschalier 2021 [12]	Prospective cohort study	France	ADHF (n = 140)	75.8 ± 10.2	58.6	24 months	1. All-cause mortality
							2. MACE
Saito 2022 [25]	Prospective cohort study	Japan	HF (n = 226)	82	51.8	1.2 years	All-cause mortality
Harada 2022 [26]	Retrospective cohort study	Japan	Chronic HF (n = 58)	72.5 ± 8.73	56.9	868 ± 617 days	MACE
Maeda 2022 [11]	Prospective cohort study	Japan	HF (n = 960)	1. Man: sarcopenia: 83	58.4	12 months	All-cause mortality
				Non-sarcopenia: 77			
				2. Woman: sarcopenia: 84			
				Non-sarcopenia: 82			

Note: MACE, major adverse cardiovascular events; HF, heart failure; HFpEF, heart failure with preserved ejection fraction; HFrEF, heart failure with reduced ejection fraction; ADHF, acute decompensated heart failure; PMI, psoas muscle mass index.

**Table 2.** **Diagnostic criteria and cutoff value used to define sarcopenia in meta-analysis**.

Study	Diagnostic criteria						
	Definition	Low muscle mass		Low muscle strength		Low physical performance	
		Cutoff value	Measure	Cutoff value	Measure	Cutoff value	Measure
Zhou 2017 [19]	AWGS	Man <7.0 kg/$m^{2}$	DXA	Man <26 kg	HG	<0.8 m/s	GS
		Woman <5.4 kg/$m^{2}$		Woman <18 kg			
Lopez 2019 [10]	Boutin	Man <5.39 ${\mathrm{cm}}^{2}$	CT	NR	NR	NR	NR
		Woman <3.66 ${\mathrm{cm}}^{2}$					
Nozaki 2019 [20]	AWGS	Man <7.0 kg/$m^{2}$	BIA	Man <26 kg	HG	<0.8 m/s	GS
		Woman <5.7 kg/$m^{2}$		Woman <18 kg			
Von Haehling 2020 [21]	EWGSOP	Man <7.26 kg/$m^{2}$	DXA	NR	NR	NR	NR
		Woman <5.45 kg/$m^{2}$					
Eschalier 2021 [12]	EWGSOP	Man <10.75 kg/$m^{2}$	BIA	Man <30 kg	HG	<0.8 m/s	GS
		Woman <6.75 kg/$m^{2}$		Woman <20 kg			
Hu 2020 [23]	CSOBMR	NR	DXA	Man <25 kg	HG	<0.8 m/s	GS
				Woman <18 kg			
Konishi 2021 [24]	AWGS	Man <7.0 kg/$m^{2}$	BIA	Man <26 kg	HG	<0.8 m/s	GS
		Woman <5.7 kg/$m^{2}$		Woman <18 kg			
Maeda 2022 [11]	AWGS	Man <7.0 kg/$m^{2}$	BIA	Man <26 kg	HG	<0.8 m/s	GS
		Woman <5.7 kg/$m^{2}$		Woman <18 kg			
Saito A 2022 [25]	AWGS	Man <7.0 kg/$m^{2}$	BIA	NR	NR	NR	NR
		Woman <5.7 kg/$m^{2}$					
Saito B 2022 [25]	EWGSOP2	Man <7.0 kg/$m^{2}$	DXA	NR	NR	NR	NR
		Woman <5.4 kg/$m^{2}$					
		Cutoff value					Measure
Onoue 2016 [18]	Ishii	Sarcopenia score:					GS, measuring tape
		Men: 0.62 × (age − 64) − 3.09 × (grip strength − 50) − 4.64 × (calf circumference − 42);					
		Women: 0.80 × (age − 64) − 5.09 × (grip strength − 34) − 3.28 × (calf circumference − 42)					
		Man: sarcopenia score ≥105; Woman: sarcopenia score ≥120					
Matsumura 2020 [22]	Takagi	Reduced PMI was defined as a PMI below the 25th sex-specific percentile according to previous reports					CT
Harada 2022 [26]	Harada	NR					CT

Note: AWGS, International Working Group on Sarcopenia; EWGSOP, European Working Group on Sarcopenia in Older People; CSOBMR, Chinese Society of Bone and Mineral Research; DXA, dual X-ray absorptiometry; HG, hand grip; GS, gait speed; CT, computed tomography; SMI, skeletal muscle index; BIA, bioelectrical impedance analysis; MMI, muscle mass index; NR, not reported; PMI, psoas muscle mass index.

### 3.3 Quality Assessment

All included studies had scores of 5 or higher on the NOS evaluation criteria, indicating high quality. One study [26] did not report the diagnostic criteria for sarcopenia. Intergroup comparability between the sarcopenic and non-sarcopenic groups was considered in three [20, 22, 24] of the twelve studies, and the other nine studies were biased in terms of intergroup comparability. Five studies [12, 18, 21, 22, 25] did not specify an appropriate follow-up time. Four studies did not clearly report the follow-up results [11, 18, 21, 25]. Table 3 (Ref. [10, 11, 12, 18, 19, 20, 21, 22, 23, 24, 25, 26]) displays the results of quality assessment.

**Table 3.** **Quality assessment of included articles**.

Study	Selection				Comparability	Exposure			Total NOS score
	Representativeness of the exposed cohort	Selection of the non-exposed cohort	Ascertainment of exposure	Demonstration that outcome of interest was not present at start of study		Assessment of outcome	Was follow-up long enough for outcomes to occur	Adequacy of follow-up of cohorts	
Onoue 2016 [18]	1	1	1	1	1	1	0	0	6
Zhou 2017 [19]	1	1	1	1	1	1	1	1	8
Nozaki 2019 [20]	1	1	1	1	2	1	1	1	9
Lopez 2019 [10]	1	1	1	1	1	1	1	1	8
Von Haehling 2020 [21]	1	1	1	1	0	1	0	0	5
Matsumura 2020 [22]	1	1	1	1	2	1	0	1	8
Hu 2020 [23]	1	1	1	1	0	1	1	1	7
Konishi 2021 [24]	1	1	1	1	2	1	1	1	9
Eschalier 2021 [12]	1	1	1	1	1	1	0	1	7
Saito 2022 [25]	1	1	1	1	0	1	0	0	5
Harada 2022 [26]	1	1	0	1	0	1	1	1	6
Maeda 2022 [11]	1	1	1	1	0	1	1	0	6

NOS, Newcastle-Ottawa Scale.

### 3.4 Prognostic Effects of Sarcopenia on Patients with HF

#### 3.4.1 Meta-Analysis of All-Cause Mortality 

**Fig. 2.**
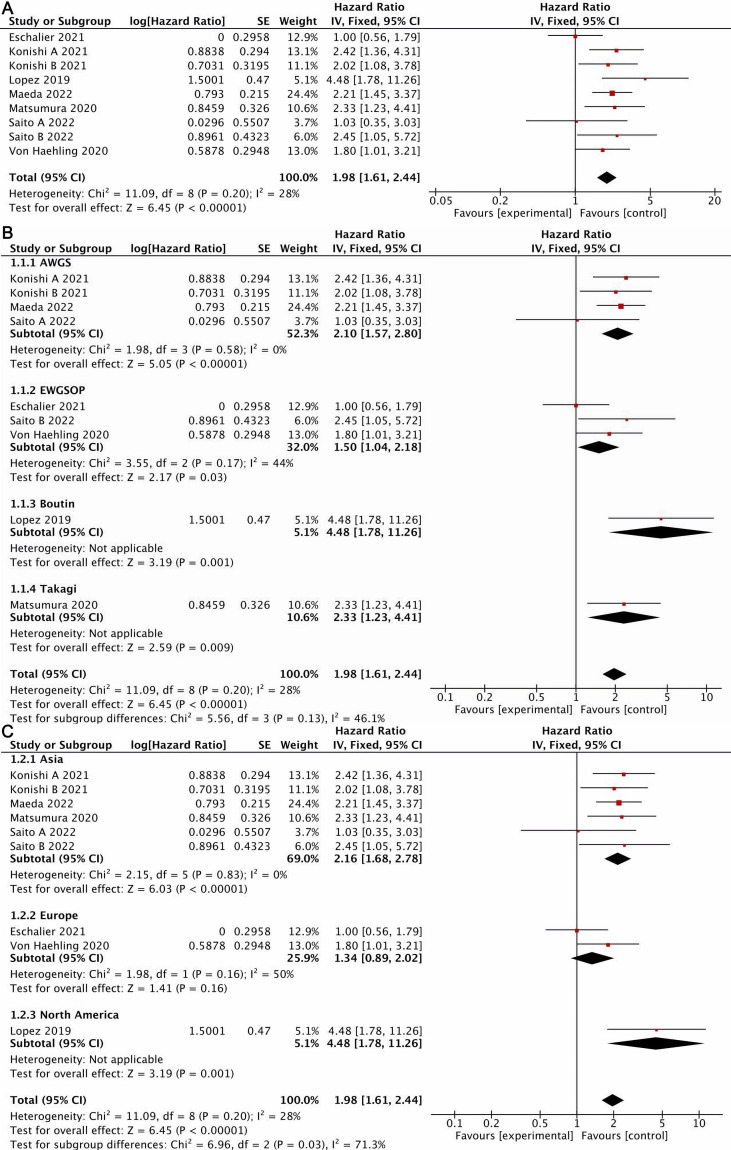
**Forest plot of all-cause mortality**. (A) All included studies. (B) Subgroup analysis by sarcopenia diagnostic criteria. (C) Subgroup analysis by region. AWGS, International Working Group on Sarcopenia; EWGSOP, European Working Group on Sarcopenia in Older People.

Nine studies [10, 11, 12, 21, 22, 24, 25] reported the effect of sarcopenia on all-cause mortality in HF patients, with low heterogeneity across the studies ($I^{2}$ = 28%, *p* = 0.20). A fixed effect model was used. The results show that there were statistically significant differences between patients with and without sarcopenia in terms of all-cause mortality (HR = 1.98, 95% CI: 1.61–2.44, *p* < 0.05) (Fig. 2A).

To investigate whether various diagnostic criteria for sarcopenia and studies from various regions have an impact on all-cause mortality, we performed a subgroup analysis. Since EWGSOP2 is an updated version of EWGSOP, they were categorized as one subgroup in the subgroup analysis. In each subgroup of the diagnostic criteria for sarcopenia, sarcopenia increased the risk of all-cause mortality in patients with HF: AWGS (HR = 2.1, 95% CI: 1.57–2.8, *p* < 0.05), EWGSOP (HR = 1.5, 95% CI: 1.04–2.18, *p* < 0.05), Boutin (HR = 4.48, 95% CI: 1.78–11.26, *p* < 0.05), and Takagi (HR = 2.33, 95% CI: 1.23–4.41, *p* < 0.05) (Fig. 2B). In terms of region subgroups, the effect of sarcopenia on all-cause mortality in patients with HF was significant in the Asian (HR = 2.16, 95% CI: 1.68–2.78, *p* < 0.05) and North American (HR = 4.48, 95% CI: 1.78–11.26, *p* < 0.05) subgroups. However, in the European subgroup, with only two studies and 408 patients, the effect of sarcopenia on all-cause mortality in HF patients was insignificant (HR = 1.34, 95% CI: 0.89–2.02, *p* > 0.05) (Fig. 2C).

Sensitivity analyses were conducted to ensure stability of the results. The sensitivity analysis showed that the results did not change significantly when each study was removed from the analysis in turn.

#### 3.4.2 Meta-Analysis of MACE

**Fig. 3.**
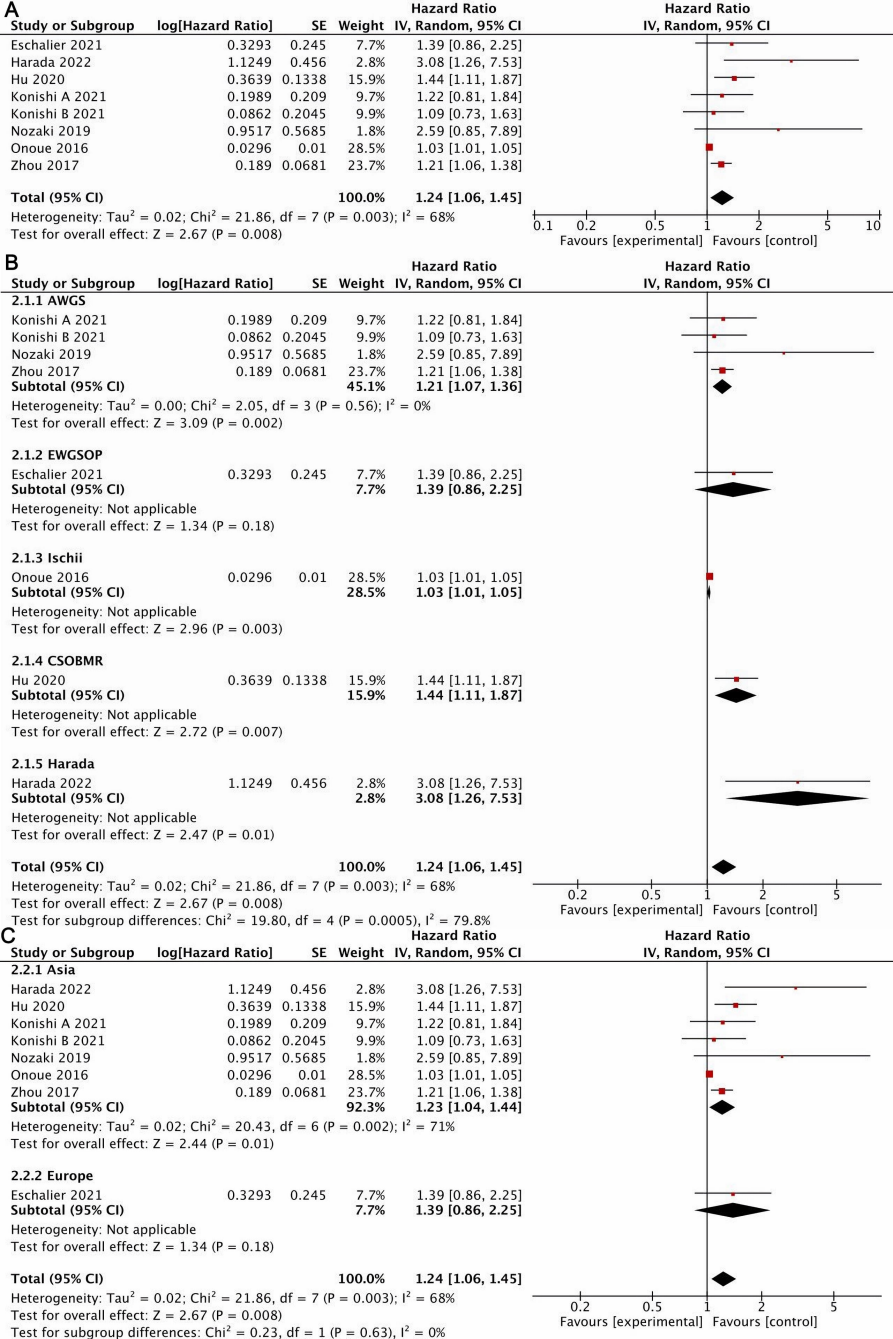
**Forest plot of MACE**. (A) All included studies. (B) Subgroup analysis by sarcopenia diagnostic criteria. (C) Subgroup analysis by region. MACE, major adverse cardiovascular events. AWGS, International Working Group on Sarcopenia; EWGSOP, European Working Group on Sarcopenia in Older People; CSOBMR, Chinese Society of Bone and Mineral Research.

Eight studies [12, 18, 19, 20, 23, 24, 26] reported the impact of sarcopenia on MACE in patients with HF, with high heterogeneity among them. A random effect model ($I^{2}$ = 68%, *p* = 0.003) was used. The results demonstrate that the risk of MACE was higher in patients with sarcopenia than in those without it, with a statistically significant difference (HR = 1.24, 95% CI: 1.06–1.45, *p* < 0.05) (Fig. 3A).

We performed subgroup analysis to determine whether various diagnostic criteria for sarcopenia and studies from diverse regions had an impact on MACE in HF patients. When considering the diagnostic criteria for sarcopenia, the results demonstrate that the association between sarcopenia and MACE was statistically significant in the AWGS (HR = 1.21, 95% CI: 1.07–1.36, *p* < 0.05), Ischill (HR = 1.03, 95% CI: 1.01–1.05, *p* < 0.05), CSOBMR (HR = 1.44, 95% CI: 1.11–1.87, *p* < 0.05) and Harada (HR = 3.08, 95% CI: 1.26–7.53, *p* < 0.05) subgroups. However, in the EWGSOP subgroup, with only one study and 140 patients, the effect of sarcopenia on MACE in HF patients was insignificant (HR = 1.39, 95% CI: 0.86–2.25, *p* > 0.05) (Fig. 3B). When considering region, the effect of sarcopenia on MACE was statistically significant in the Asian subgroup (HR = 1.23, 95% CI: 1.04–1.44, *p* < 0.05). However, in the European subgroup, with only 1 study and 140 patients, the effect of sarcopenia on MACE in HF patients was nonsignificant (HR = 1.39, 95% CI: 0.86–2.25, *p* > 0.05), as shown in Fig. 3C.

In the sensitivity analysis, the results did not change significantly when each study was removed from the analysis sequentially.

## 4. Discussion

This meta-analysis sought to determine whether sarcopenia might be used to forecast prognosis in HF patients and if different diagnostic criteria for sarcopenia and various regions affect prognostic outcomes. The results of 12 cohort studies with 3696 participants revealed that the sarcopenia population had a greater incidence of MACE and all-cause death. However, due to insufficient original literature, the findings in the diagnostic criteria and region subgroups still need further validation.

Our findings revealed a higher risk of all-cause mortality and MACE in HF patients with sarcopenia than those without, which is similar to the results of previous studies. Previous meta-analyses have shown the role of sarcopenia on the prognosis of cardiovascular disease. Xue [34] studied the prognostic value of sarcopenia in elderly patients with coronary artery disease and showed that sarcopenia was associated with adverse cardiovascular events. Dakis [35] investigated the relationship between sarcopenia and prognosis in patients undergoing endovascular aortic aneurysm repair, and the study showed that sarcopenia was associated with worse long-term survival. Although no previous meta-analysis explored the association between sarcopenia and HF prognosis, there are original studies that have attempted to verify the existence of the correlation, albeit using different screening methods for sarcopenia. Katano [36] used the skeletal muscle index (SMI) predicted from anthropometric indicators as a screening tool for sarcopenia. Cunha [37] treated pectoralis muscle size as an assessment tool for sarcopenia. The results both showed that sarcopenia was associated with poor prognosis in HF patients, which is consistent with the findings of this study.

Previous studies have reported that sarcopenia leads to increased all-cause mortality [36] and MACE rates [38], resulting in poor prognosis in patients with HF. Sarcopenia is one of the leading causes of exercise intolerance and ventilatory inefficiency in patients with HF, which worsens the clinical status of HF, leading to longer hospital stays, frequent readmissions, decreased quality of life, and poor prognosis [39, 40, 41, 42]. Studies have also shown that HF can complicate the progression and outcome of sarcopenia. On the one hand, reduced cardiac output, decreased food intake, and lowered exercise capacity in HF patients promote the release of inflammatory factors, increase sympathetic excitability, and affect the secretion of muscle-related hormones. These factors act together in muscle tissue, leading to skeletal muscle atrophy [43]. On the other hand, HF can exacerbate adverse outcomes associated with sarcopenia, including falls, osteoporosis, weakness, hospitalizations and mortality [8]. A recent meta-analysis [44] showed that the overall pooled prevalence of sarcopenia was 34% in patients with HF, reminding us that sarcopenia should be specifically considered in patients with HF. Given the hazards and prevalence of sarcopenia, early detection of the functional status of sarcopenia in clinical practice is crucial for effective identification and timely intervention in HF patients with poor prognosis.

Multiple diagnostic criteria for sarcopenia were used in the studies. Therefore, we conducted a subgroup analysis to verify whether the different diagnostic criteria affect the prognostic outcome of patients with HF. The study results showed that in the subgroup analysis of all-cause mortality, all subgroups were statistically different. In the subgroup analysis of MACE, the AWGS subgroup was statistically different, while the EWGSOP subgroup was not. However, as only one study was included in the EWGSOP subgroup of MACE, the current conclusion needs to be validated by incorporating more studies. The reason why there is no statistical difference may be that the limited sample size affects research results and the robustness of conclusions. The importance of sarcopenia cannot be ignored, but there is still no consensus on cutoff values in defining sarcopenia [45]. Even with the same version of the diagnostic criteria and using the same measurement tools, the cutoff value of sarcopenia is different when the calculation criteria is different, which poses a challenge to early identification and timely intervention of sarcopenia [46]. In addition, according to EWGSOP, diagnostic criteria for sarcopenia should include muscle mass, muscle strength, and physical performance [29]. However, patients with some special conditions, such as arm or leg fractures, are unable to measure muscle strength and physical performance, which makes it difficult for the diagnosis and treatment of sarcopenia [44]. Therefore, this kind of situation should be taken into account when updating the consensus of sarcopenia in the future, thus contributing to the clinical application and promotion of sarcopenia diagnosis and treatment.

People in different regions may have varying lifestyles and physical activity levels due to ethnic and environmental factors, which could influence body composition [47]. This paper includes original studies from different regions were included in this paper, and a subgroup analysis was conducted to verify whether the region influenced the role of sarcopenia on the prognosis of HF. The results of the study showed that for all-cause mortality, there was a statistical difference between the Asian and North American subgroups, but no statistical difference in the European subgroup. For MACE, there was a statistical difference in the Asian subgroup, but no statistical difference in the European subgroup. The lack of statistical significance in European subgroups for all-cause mortality and MACE does not necessarily imply that sarcopenia has no prognostic effect on HF in Europe. This could be due to the limited number of original studies, which have not found statistical differences for the time being. This should be interpreted with caution when explaining the conclusions in order to avoid bias and affect the generalization and application of the conclusions. Tantai [48] found a higher risk of mortality in the European subgroup of cirrhosis patients with sarcopenia. Xu [49] discovered that sarcopenia is associated with mortality in adults, which is inconsistent with our findings. Considering the non-robustness of the findings in this study and the variability of the conclusions with other studies, there is still a growing need to incorporate more original studies from Europe in the future to confirm and update the current conclusions of our study.

This meta-analysis has several limitations that should be noted. First, the included studies used different diagnostic criteria for sarcopenia and cutoff values were used in included studies, which may have contributed to the heterogeneity of the study. A universally agreed-upon diagnostic criterion for sarcopenia is needed, and the cutoff value should be adjusted for race, gender, and age to account for demographic variables while facilitating the diagnosis and treatment of sarcopenia [50]. Second, many studies used bioelectrical impedance analysis (BIA) to assess muscle mass, but due to fluid overload in patients with HF, muscle mass may be overestimated [51]. Third, due to limited data, the findings of diagnostic criteria and region subgroups need to be verified by including more literature, and the conclusion should be treated with caution. Fourth, only English and Chinese literature was included, which may be subject to publication bias. However, funnel plots and Egger tests were not used to assess possible publication bias because the number of studies included in each subgroup was less than 10, in which case funnel plots and Egger tests could produce misleading results [52, 53]. Additionally, the included studies were from different regions, with diverse healthcare systems and various medical technologies, which could limit the generalizability of the results [54]. In the future, we hope that more countries and regions will pay attention to the prognostic effect of sarcopenia on HF patients and conduct more high-quality studies, thus enriching and updating the conclusions of this paper and promoting the generalizability of the findings.

## 5. Conclusions

Sarcopenia is associated with a poor prognosis in patients with HF, including all-cause mortality and MACE. However, due to insufficient data, the results of the diagnostic criteria and region subgroups still need further validation through the inclusion of more studies. To better validate the association between sarcopenia and poor prognosis in patients with HF, future studies should test this association with different diagnostic criteria for sarcopenia adopted in diverse regions of the world. Therefore, caution should be exercised when interpreting this part of the findings. Our study supports the value of screening for sarcopenia in patients with HF, which may provide an initial basis for early identification and prediction of poor prognosis.

## Data Availability

Not applicable.

## Supplementary Material

Supplementary material associated with this article can be found, in the online version, at https://doi.org/10.31083/j.rcm2409273.
