# Supplementary material for: Sarcopenia Predicts Adverse Prognosis in Patients with Heart Failure: A Systematic Review and Meta-Analysis
Source: Rev Cardiovasc Med. 2023 Sep 25;24(9):273. doi: 10.31083/j.rcm2409273 (PMC11270102; doi:10.31083/j.rcm2409273)
Supplement: Supplementary file 1 [file 2153-8174-24-9-273-s1.zip › Supplementary Material 2 Search Strategies.docx]

**Search Strategies（**Search Date: March 2023**）**

**PubMed**

#1 sarcopenia [MeSH Terms]. (8,774)

#2 sarcopenia [Title/Abstract] OR sarcopeni* [Title/Abstract] OR muscle weakness [Title/Abstract] OR muscle atrophy [Title/Abstract] (41,963)

#3 #1 OR #2 (42,697)

#4 heart failure [MeSH Terms] (144,654)

#5 heart failure [Title/Abstract] OR HF [Title/Abstract] OR cardiac failure [Title/Abstract] OR heart decompensation [Title/Abstract] OR myocardial failure [Title/Abstract] OR congestive heart failure [Title/Abstract] (253,197)

#6 #4 OR #5 (287,340)

#7 #3 AND#6 (1033)

**Cochrane**

#1 MeSH descriptor: [Sarcopenia] explode all trees (742)

#2 (sarcopenia):ti,ab,kw OR (sarcopeni*):ti,ab,kw OR (muscle weakness):ti,ab,kw OR (muscle atrophy):ti,ab,kw (8,662)

#3 #1 OR #2 (8,662)

#4 MeSH descriptor: [Heart Failure] explode all trees (12,391)

#5 (heart failure):ti,ab,kw OR (HF):ti,ab,kw OR (cardiac failure):ti,ab,kw OR (heart decompensation):ti,ab,kw OR (myocardial failure):ti,ab,kw OR (congestive heart failure):ti,ab,kw (48,726)

#6 #4 OR #5 (48,748)

#7 #3 AND #6 (318)

**Embase**

#1 'sarcopenia'/exp (18,824)

#2 'sarcopenia':ab,ti OR 'sarcopeni*':ab,ti OR 'muscle weakness':ab,ti OR 'muscle atrophy':ab,ti (61,124)

#3 #1 OR #2 (64,282)

#4 'heart failure'/exp (636,005)

#5 'heart failure':ab,ti OR 'HF':ab,ti OR 'cardiac failure':ab,ti OR 'heart decompensation':ab,ti OR 'myocardial failure':ab,ti OR 'congestive heart failure':ab,ti

(392,938)

#6 #4 OR #5 (731,753)

#7 #3 AND #6 (2,487)

**Chinese National Knowledge Infrastructure(CNKI)**

篇关摘：（肌少症 OR 少肌症 OR 骨骼肌减少症） AND 篇关摘：（心力衰竭 OR 心脏衰竭 OR 心衰） (74)
